# Liquid biopsy in metastatic breast cancer

**DOI:** 10.20517/cdr.2019.84

**Published:** 2019-12-19

**Authors:** Malgorzata Banys-Paluchowski, Peter Paluchowski

**Affiliations:** ^1^Department of Gynecology and Obstetrics, Asklepios-Klinik Hamburg Barmbek, Hamburg 22307, Germany.; ^2^Department of Gynecology and Obstetrics, Regio Klinikum Pinneberg, Pinneberg 25421, Germany.

**Keywords:** Tumor cell dissemination, circulating tumor cell, liquid biopsy, circulating tumor DNA, breast cancer

## Abstract

The spread of single tumor cells shed by the primary tumor has been observed in most solid carcinomas and is generally associated with poor clinical outcome. Tumor cells detected in the peripheral blood are commonly referred to as circulating tumor cells (CTCs) and are seen as possible precursors of metastatic disease. Beyond CTCs, circulating tumor DNA and non-coding RNA are increasingly the focus of translation cancer research. In metastatic breast cancer (MBC), elevated levels of CTCs have been confirmed as an independent prognostic factor. While detection of elevated counts after the start of systemic therapy predicts poor response, it is unclear which treatment strategy should be offered in the case of CTC persistence. Currently, the main potentials of blood-based diagnostics in BC are therapy monitoring and liquid biopsy-based treatment interventions. Recently, the first positive study on CTC-guided therapy choices in hormone receptor positive HER2 negative MBC was published. In the present review, we discuss the current data and potential clinical application of liquid biopsy in the metastatic setting.

## Introduction

Hematogenous spread of isolated tumor cells in a patient with a solid tumor was first described in 1869 by the Australian physician Thomas Ashworth^[1]^. Since then, the possibilities of blood-based diagnostics have been extensively studied and the accumulated body of evidence has with time challenged the previously accepted view of breast cancer (BC) as a localized disease that requires an ultraradical surgical approach. Currently, it is widely accepted that single tumor cells encountered in peripheral blood [circulating tumor cells (CTCs)] and bone marrow [disseminated tumor cells (DTCs)] are precursors of metastatic growth and are sometimes described as minimal residual disease in the adjuvant setting. We and others have previously shown that these cells may be detected even in patients with the earliest stages of disease or pre-invasive lesions^[2,3]^. In patients with early BC, the detection of CTCs and DTCs identifies patients most at risk for a relapse and death from BC^[4,5]^. Recently, two large clinical trials have investigated the role of CTC persistence 5 years after diagnosis in asymptomatic patients^[6-8]^. Both have demonstrated that women with detectable CTCs have higher risk of recurrence in the following years; this association was highly significant in patients with hormone receptor (HR)-positive cancer.

In metastatic breast cancer (MBC), several clinical applications of blood-based diagnostics have been explored: (1) prediction of the clinical outcome (prognostication); (2) prediction of response to treatment (therapy monitoring); and (3) liquid biopsy-guided treatment decisions.

In this review, we discuss the current data on liquid biopsy and the clinical relevance of CTCs and circulating tumor DNA (ctDNA) in MBC.

## Prognostication

In 2004, Cristofanilli *et al*.^[9]^ reported that elevated CTC counts in women with MBC predict shorter progression-free and overall survival (PFS and OS, respectively). In this study, a standardized immunocytochemistry-based assay called CellSearch was used to detect CTCs^[10]^. Since the numbers of detectable tumor cells in MBC are generally much higher than those in early disease, five CTCs in 7.5 mL blood served as a cut-off to discriminate between patients with favorable and poor outcome. Since then, the CellSearch detection system has been approved by the FDA and is currently used in the majority of clinical trials^[11]^. Recently, a large pooled analysis of individual patient data from 18 centers has confirmed elevated CTC counts as an independent predictor of shorter PFS, OS, and BC specific survival^[12]^. Most importantly, contrary to some previous reports^[13]^, the association with clinical outcome was independent of the tumor subtype.

The evidence concerning the clinical significance of ctDNA is less clear. In 1977, Leon *et al*.^[14]^ reported significantly higher DNA levels in the blood of patients with metastatic disease, as compared to non-metastatic patients. A large proportion of circulating DNA fragments encountered in the peripheral blood is shed by dying or necrotic tumor cells^[15]^. However, DNA fragments may be detected in blood samples of healthy subjects as well, usually not exceeding concentrations of 10 ng/mL^[15]^. Therefore, ctDNA accounts for only a small fraction of free circulating DNA and the presence of circulating DNA is not tumor-specific^[16]^. Bettegowda *et al*.^[17]^ reported that ctDNA was detectable in > 75% of patients with advanced breast carcinoma, compared to 50% of those with localized tumors. Recently, a large meta-analysis including 10 studies examined the prognostic relevance of cell-free DNA (cfDNA) in 1127 patients with BC^[18]^. Six studies examined only patients with early BC (stages I-III, *n* = 840), two studies included metastatic patients (stage IV, *n* = 183), and two studies included both groups (stages I-IV, *n* = 104). The pooled hazard ratio showed significant correlations between cfDNA and OS (HR = 2.41, 95%CI: 1.83-3.16) and disease-free and relapse-free survival (DFS and RFS, respectively) (HR = 2.73, 95%CI: 2.04-3.67), thus confirming the prognostic potential of circulating DNA.

## Therapy monitoring

Studies on early BC have shown that CTCs are able to persist beyond (neo)adjuvant chemotherapy in a large proportion of patients. One of the mechanisms enabling these cells to elude cytotoxic treatment is probably their ability to enter a dormant low-proliferative state for a prolonged period of time. In metastatic BC, several studies have investigated the dynamics of CTC numbers under therapy. Cristofanilli *et al*.^[9]^ reported that women with elevated CTC counts at first follow up visit (approximately 3 to 4 weeks after initiation of therapy) relapsed sooner than those whose CTC numbers fell under the cutoff of five CTCs per 7.5 mL blood (2.1 months *vs*. 7.0 months; *P* < 0.001); their overall survival was significantly shorter as well.

**Figure 1:**
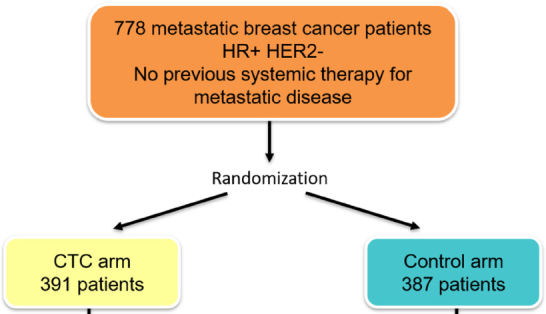
Design of the SWOG 0500 trial^[19]^

The optimal strategy in these patients was addressed by the SWOG 0500 trial^[19]^ [Figure 1]. This study included 595 patients with MBC starting first-line chemotherapy in whom CTCs were measured using the CellSearch system. Only patients with initially elevated CTC counts were included in the further study. After the first cycle of therapy, 123 patients with persistent high CTC numbers were randomized to continue the same chemotherapy or to switch to an alternative regimen. However, the switching strategy failed to improve patient outcomes suggesting that persistently increased CTCs after start of first-line chemotherapy may identify patients who are resistant to several commonly used chemotherapeutic agents. Whether these patients might benefit from experimental, immunotherapeutic, or targeted approaches remains to be clarified in further studies.

With regard to therapy monitoring using ctDNA, a proof-of-concept study was presented in 2013 by Dawson *et al*.^[20]^. ctDNA, CTCs, and tumor marker CA 15-3 were measured in the blood of 30 MBC patients receiving systemic therapy. Levels of ctDNA showed a greater dynamic range and stronger correlation with changes in tumor burden than did CA 15-3 or CTCs. Furthermore, ctDNA provided the earliest assessment of response to therapy in 10 out of 19 patients (53%).

## Liquid biopsy-guided treatment decisions: are we ready yet?

Beyond prognostication and therapy monitoring, blood test-guided therapies have emerged as the real “Holy Grail” of the liquid biopsy-based research in recent years. In other entities, such as non-small-cell lung cancer (NSCLC), treatment indications can already be based on alterations found in plasma samples; e.g., the kinase inhibitor targeting epidermal growth factor receptor (EGFR) erlotinib is available for NSCLC patients whose tumors have EGFR exon 19 deletions or exon 21 (L858R) substitution mutations, detected either in the circulating DNA or in tumor tissue. Thus, in the case of progression, an invasive lung biopsy may be substituted by simple blood sampling in a large proportion of patients.

In BC, extensive research has been conducted on the usefulness of CTCs and ctDNA for guiding treatment decisions, leading to the first liquid biopsy-based FDA approval in MBC in May 2019. The approval of the oral PI3K inhibitor alpelisib was based on the phase III double-blind SOLAR-1 trial^[21]^. MBC patients with hormone receptor (HR)-positive HER2-negative tumors who relapsed following endocrine therapy were randomized to fulvestrant and placebo or fulvestrant and alpelisib. Approximately 40% of HR-positive BC patients have PIK3CA mutations, leading to activation of the PI3K pathway and resistance to endocrine therapy. In the SOLAR-1 trial, the PIK3CA mutation status was primarily assessed in the tumor tissue. In patients with PIK3CA-mutated cancer, PFS was significantly longer in the alpelisib arm (11.0 months *vs*. 5.7 months; hazard ratio for progression or death: 0.65, 95%CI: 0.50-0.85). In addition, ctDNA in plasma samples was analyzed in a subgroup of patients. Interestingly, PIK3CA mutational status in the blood significantly predicted PFS benefit as well (hazard ratio: 0.55, 95%CI not available)^[22]^. Based on these data, the FDA approved alpelisib for patients with mutations in cfDNA and/or tumor tissue. The approval in the European Union is still outstanding.

Furthermore, the identification of patients at risk for developing drug resistance is one of the potential areas of interest in the context of ctDNA-based therapies. Among somatic mutations acquired by tumor cells, the ESR1 mutations are the focus of increasing interest as they may lead to subsequent estrogen-independent transcriptional activity. Turner *et al*.^[23]^ randomized 693 patients with advanced BC after failure of endocrine therapy with non-steroidal aromatase inhibitors to fulvestrant *vs*. exemestane. In the overall population, no differences regarding time to progression of overall response rates were observed. However, patients with circulating ESR1 mutations were more likely to benefit from fulvestrant (PFS 3.5 *vs*. 2.0 months). In the subsequent meta-analysis of the EFECT and SoFEA trials, patients with ESR1-mutant ctDNA receiving exemestane had the poorest clinical outcome, suggesting that blood-based mutational analysis may identify patients not likely to benefit from aromatase inhibitor therapy.

ESR1 dynamics have been further explored in a translational subgroup analysis of the PALOMA 3 trial, one of the studies investigating CDK4/6 inhibitors in HR-positive HER2-negative MBC^[24]^. In this trial, patients who relapsed following endocrine therapy were randomized to fulvestrant with palbociclib *vs*. fulvestrant with placebo. O’Leary *et al*.^[24]^ conducted paired baseline and end of treatment ctDNA sequencing from 195 patients enrolled in the study and showed that clonal evolution is a frequent event observed during treatment. For instance, RB1 mutations emerged only in the palbociclib plus fulvestrant arm and in a minority of patients. New driver mutations emerged in PIK3CA and ESR1 after treatment in both arms. Interestingly, evolution of driver mutations was uncommon in patients with early progression on palbociclib plus fulvestrant but common in patients progressing later during treatment.

With regard to patients with triple-negative MBC, copy number alterations (CNA) measured in cfDNA emerged as a promising biomarker. Stover *et al*.^[25]^ performed genome-wide sequencing of cfDNA from plasma from 164 patients with triple-negative tumors and showed that 18q11 and 19p13 gains in CNA were associated with metastatic survival that was independent of clinicopathologic factors^[25]^. Potentially, gain or ampliﬁcation of both regions in the blood sample may identify a subset of triple-negative rapid progressors with remarkably poor survival.

**Figure 2:**
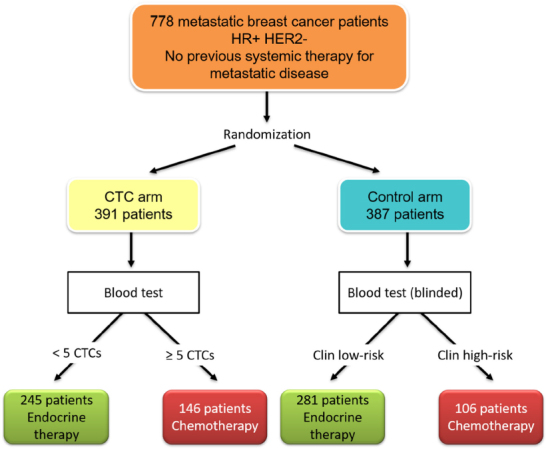
Study design of STIC circulating tumor cells (CTCs) initiated by the Intitut Curie in Paris^[26]^

With regard to CTCs, the first positive trial on blood-based treatment interventions was presented at the San Antonio BC Symposium in 2018^[26]^. The STIC CTC trial was initiated by the French Insitut Curie and investigated the optimal first-line therapy for HR-positive HER2-negative MBC patients [Figure 2]. After randomization, patients in the control arm received therapy of physician’s choice: chemotherapy in the case of a clinically high-risk disease or endocrine monotherapy if the tumor was classified as clinically low-risk. In the CTC arm, the therapy was chosen according to the results of the CellSearch blood test: patients with < 5 CTCs per 7.5 mL blood received endocrine therapy, while those with ≥ 5 CTCs were given chemotherapy. After a median follow up of 30 months, the PFS and OS in both groups was identical. Interestingly, patients with a discordant status (e.g., clinically high-risk but with < 5 CTCs or clinically low-risk and ≥ 5 CTCs) benefited from chemotherapy: both the PFS and OS were significantly longer if chemotherapy was administered (*P* = 0.001 for PFS and *P* = 0.04 for OS). Thus, it could be shown for the first time that CTC-based therapy intervention led to better clinical outcome. However, it is difficult to translate these results into the clinical routine, since new substances, most importantly the CDK4/6 inhibitors, have been approved for treatment of HR-positive HER2-negative MBC after the initiation of the STIC CTC trial and nowadays more than 60% of this subgroup receive endocrine-based combination therapy in the first-line setting^[27]^.

**Figure 3:**
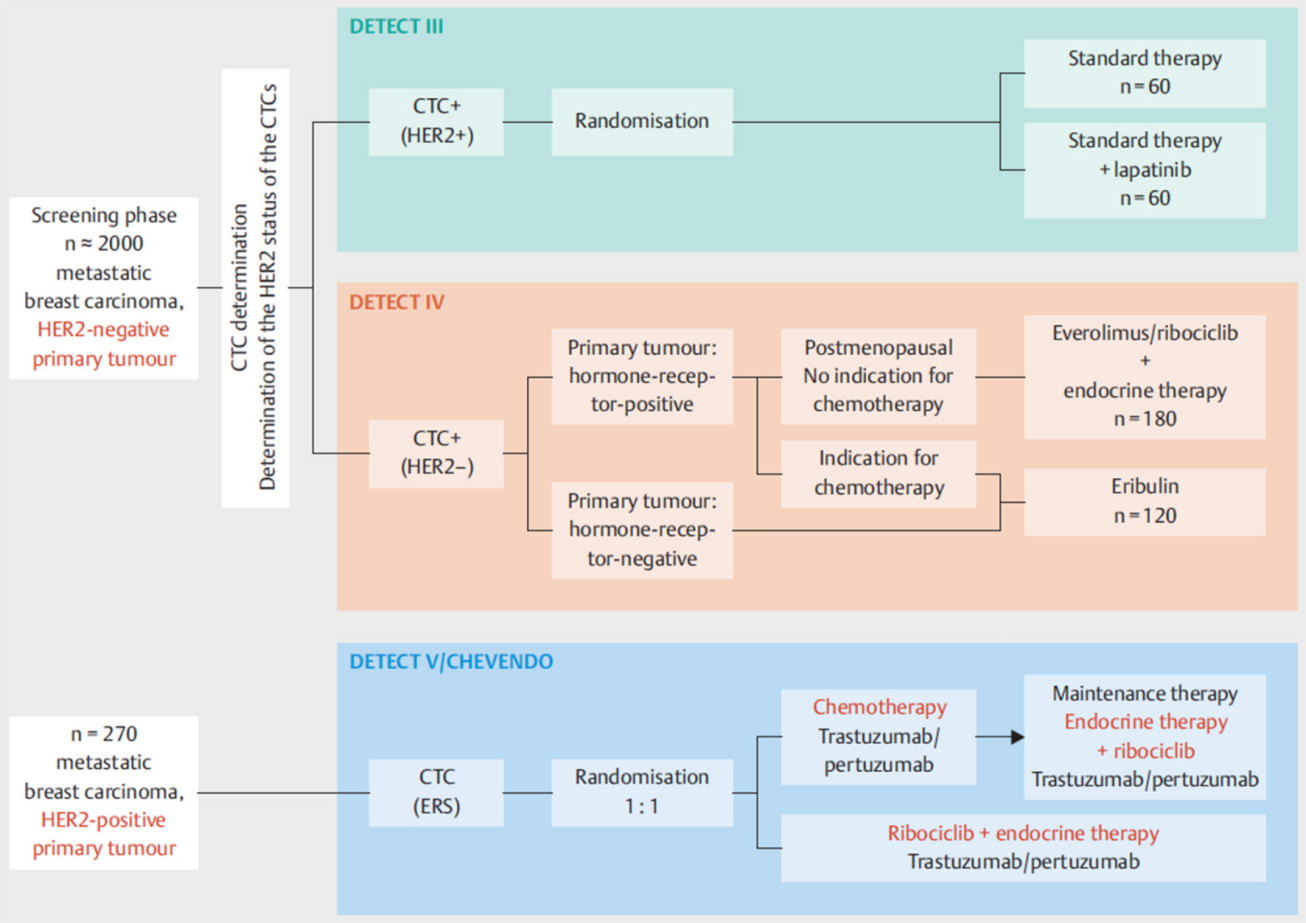
Design of the studies within the DETECT trial program - the largest study program on circulating tumor cell (CTC)-guided therapies worldwide^[11]^

Other currently recruiting trials focus on the characteristics of CTCs rather than their numbers. Most notably, the German DETECT study program aims at establishing a possible benefit of targeted therapies chosen according to the receptor status of CTCs and not primary tumor or metastasis [Figure 3]. Previous data have shown that CTCs frequently differ from cells in the primary tumor or metastasis with regard to prognostic and predictive markers^[28-30]^. In the phase III DETECT III trial, patients with HER-negative MBC are screened for HER2-positive CTCs and, if such cells are detected, they are randomized to standard therapy ± anti-HER2 therapy lapatinib. Patients with HER2-negative CTCs are available for DETECT IVa and IVb trials and those with HER2-positive MBC can participate in the phase III DETECT V study.

## Conclusion

In the last decades, blood-based diagnostics have become one of the major focuses of oncological and translational research. Elevated levels of CTCs and tumor DNA serve as an important prognostic factor in metastatic BC and can complement therapy monitoring. Beyond enumeration, assessment of mutational status of ctDNA and phenotypic features of CTCs holds great promise in terms of liquid biopsy-guided treatment interventions. In 2019, the SOLAR-1 trial led to the first liquid biopsy-based approval in MBC in the history of the FDA. Furthermore, the French STIC CTC trial has become the first study on liquid biopsy-based interventions to demonstrate that enumeration of CTCs may guide treatment decisions in metastatic BC.

## Declarations

### Copyright

© The Author(s) 2019.
